# Evaluation of Wearable Sensor Devices in Parkinson's Disease: A Review of Current Status and Future Prospects

**DOI:** 10.1155/2020/4693019

**Published:** 2020-09-23

**Authors:** Ruirui Lu, Yan Xu, Xiaohui Li, Yongli Fan, Weiqi Zeng, Yang Tan, Kang Ren, Wenwu Chen, Xuebing Cao

**Affiliations:** ^1^Department of Neurology, The First Affiliated Hospital of Henan University, Kaifeng 475001, China; ^2^Department of Neurology, Union Hospital, Tongji Medical College, Huazhong University of Science and Technology, Wuhan 430022, China; ^3^GYENNO Technologies Co., Ltd., Shenzhen 518000, China

## Abstract

Parkinson's disease (PD) decreases the quality of life of the affected individuals. The incidence of PD is expected to increase given the growing aging population. Motor symptoms associated with PD render the patients unable to self-care and function properly. Given that several drugs have been developed to control motor symptoms, highly sensitive scales for clinical evaluation of drug efficacy are needed. Among such scales, the objective and continuous evaluation of wearable devices is increasingly utilized by clinicians and patients. Several electronic technologies have revolutionized the clinical monitoring of PD development, especially its motor symptoms. Here, we review and discuss the recent advances in the development of wearable devices for bradykinesia, tremor, gait, and myotonia. Our aim is to capture the experiences of patients and clinicians, as well as expand our understanding on the application of wearable technology. In so-doing, we lay the foundation for further research into the use of wearable technology in the management of PD.

## 1. Introduction

Parkinson's disease (PD) is a chronic progressive degenerative disease affecting the central nervous system (CNS) [1]. It was first described in 1817 by *James Parkinson* [2–6]. Impaired motor function is one of the characteristics of PD. And the motor symptoms include bradykinesia, tremor, myotonia, and postural balance disturbance and are the basis for diagnosis. In addition, sleep disorders, mental disorders, autonomic dysfunction, and other nonmotor symptoms are also used as secondary evidence of PD diagnosis [7–9]. Epidemiological data shows that the incidence of PD is 0.1-0.2% and is on the rise globally [7]. Currently, PD is the second most common neurodegenerative disease.

In the clinic, the severity of PD motor symptoms and nonmotor symptoms were mainly evaluated by a scale [10] and family diaries [11]. Research has shown that such scales are subjective and yield inconsistent results, with significant biases [12]. Due to the lack of specific diagnostic biomarkers, it is currently challenging to implement early and effective diagnosis of PD [1, 13, 14]. A meta-analysis study [8] to evaluate the accuracy of clinical diagnosis of PD reported in the last 25 years showed that the diagnostic accuracy by nonexperts was 73.8% and the diagnostic accuracy by the movement disorder specialists increased from 79.6% in the initial diagnosis to 83.9% in the follow-up diagnosis. The diagnostic criteria of the International Parkinson and Movement Disorder Society are more sensitive and specific compared to those of the United Kingdom Brain Bank [9]. However, the accuracy of the diagnostic criteria of the International Parkinson and Movement Disorder Society is not satisfactory, as it may yield some level of misdiagnosis. Severe PD is associated with more intense motor symptoms and nonmotor symptoms, which inevitably lead to motor complications [13] such as symptom fluctuation and dyskinesia. Therefore, early diagnosis and treatment are of great significance to the effective management of PD.

To fully understand the clinical manifestations and prognosis of PD, accurate and objective evaluation tools are urgently needed. Wearable devices have been widely used in PD management. This is because they are highly objective, accurate, and sustainable. In this review, we analyze the reliability of wearable technology in real-time monitoring of tremor [2, 15–22], frozen gait [23–33], movement disorder [18, 34–39], and rigidity [40] in experimental and familial environment. We provide the experimental basis for further exploration into the kinematics mechanism of PD. This will also enhance early and differential diagnosis [41, 42], in addition to providing strategies of improving the management of PD. The shortcomings and prospects of current evaluation tools are also described.

## 2. Wearable Sensors

Wearable sensors are portable and movable accessories that can be worn on the body or embedded in clothes. These devices include smart glasses, smart watches, smart clothes, or pressure shoes, among others. They contain both special hardware and software technology, with unique functions of collecting spatiotemporal kinematic parameters, data processing, storage, and transmission. With the rapid development of the Internet technology and the advent of machine learning, wearable technology has been widely used in various fields. Currently, most of the wearable devices used in the field of PD are gyroscopes, accelerometers, or magnetometers [22, 35, 43–47]. Through specific motion programs, these devices can execute real-time monitoring of PD motion symptoms for establishing multiple data models. This enables doctors to accurately analyze the patients' motion state in real time.

## 3. Current Status of Wearable Sensors in the Evaluation of Movement Symptoms in PD

Research has proved that wearable devices are as effective as the standard scale scores in evaluating PD symptoms [25]. These devices overcome the limitations associated with clinical evaluation scales in terms of objectivity, accuracy, and sensitivity. Notably, the accuracy of assessment scales is limited by the subjectivity and professional knowledge of the assessing physician and the emotion, compliance, and recall bias of patients [48]. The wearable technology improves the accuracy of evaluation by objectively quantifying sports symptoms. Besides, the wearable devices are highly sensitive, thus being able to detect subtle motor abnormalities [22], enabling early [49] or differential [50] diagnosis, and monitoring changes of motion state [51], and long-term and long-distance monitoring of motion [19]. Clinically, rapid and instantaneous assessment may not reveal the true severity of symptoms or fully reflect the state of patients in daily life. Thus, wearable devices provide clinicians with a comprehensive report of the status of patients in a single assessment, thus optimizing treatment plans. In addition, the use of wearable devices is not limited by time or place. These devices are therefore ideal for telemedicine, conforming to the future needs of the medical industry. Application of these devices transforms the medical care from being centralized clinical care to individualized diagnosis and treatment.

## 4. Bradykinesia

Bradykinesia is a typical and common symptom of PD. During bradykinesia, most patients show slowness of movement at onset, or experience reduced spontaneous movement, slow and clumsy, as well as reduced speed or amplitude of movement during rapid and repetitive movements [52]. Bradykinesia significantly impairs the quality of life of patients. Often, physicians use subtasks of the Unified Parkinson's Disease Rating Scale (UPDRS) [53, 54] scoring system to assess the severity of bradykinesia on a scale of 0 to 4 [21, 52, 54] (0: normal; 1: slight; 2: mild; 3: moderate; and 4: severe). Another commonly used method is Timed Up and Go (TUG) [55–59], which is a comprehensive test for evaluating the motion state of the patients, such as standing up, walking, turning, or sitting down. Besides, doctors use the asymmetry of swing arm as a basis for evaluation. In PD patients, the swing speed and amplitude of the more affected side are significantly reduced, which is a special symptom of bradykinesia at the clinical level [55]. However, all these semiquantitative but subjective evaluation methods are helpful for monitoring motor retardation, their evaluation results may be biased according to individual experience and expertise.

In this study, the use of wearable technology such as magnetic sensors, electromagnetic sensors, touch sensors, gyroscopes, and accelerometers [53, 54, 60, 61] to evaluate and monitor bradykinesia in PD patients is described. Studies [52] have shown that the result obtained from wearable sensors having gyroscopes or accelerometers are proportional to those recorded by experienced neurologists. Moreover, compared with standardized tests, wearable devices have high specificity (88%) and sensitivity (95%) in measuring motion retardation [25]. Unlike standard evaluation methods which are limited in diagnosing early-stage PD, wearable devices can identify early-stage PD symptoms.

In a study [56] testing the performance of an automated TUG that uses portable inertial sensors to detect and separate subcomponents of TUG test, it was found that this device could effectively diagnose early PD symptoms based on motor parameters such as cadence, angular velocity of arm-swing, turning duration, and time to perform turn-to-sits. In subsequent studies, Espay et al. [54] observed that the decline of amplitude is more obvious than speed and rhythm, and the speed could achieve complete clinical remission after taking dopamine. Interestingly, the remission of bradykinesia was more significant than dyskinesia. However, none of the above studies identified the most important parameter for assessing bradykinesia. It is therefore necessary to determine the most accurate and specific parameter that can be used as an indicator of bradykinesia.

Contrary to previous research conducted in laboratory conditions, a study by Synnott et al. [61] showed that the application of Nintendo Wii Remote enhanced objective 15-day assessment of the severity of movement symptoms of PD patients at home. Elsewhere, Memedi et al. [36] reported that a touchscreen telemetry device was able to quantify motor symptoms during episodes and peak dose dyskinesia of advanced PD patients. This study demonstrated that the device could objectively quantify PD-specific and treatment-induced motor complications. With the continuous development of science and technology, highly sensitive wearable devices will be developed to promote PD research. Previously, Ferraris et al. [62] used an optical RGB-Depth to perform automated assessment of posture and motor tasks using a self-managed and home-based approach. They revealed that the wearable devices could efficiently perform automated assessment of PD patients at home. These findings are fundamental to the design of a telemedicine framework encompassing teleneuromonitoring and neurorehabilitation. However, the patient selection process was not random in this study; thus, bias cannot be ruled out. Hence, more studies are required to improve the application of wearable devices in the future.

## 5. Tremor

Tremor is often the earliest occurrence of Parkinson's disease. It usually begins from one side of the upper limb, into the ipsilateral lower limb, and then spreads to the contralateral upper and lower limbs. It happens during rest, relaxes during voluntary movement, intensifies during nervous excitement, and disappears during sleeping. A rub-pill like involuntary movement, with a frequency of 4–6 Hz, typically characterizes the tremor [16]. Whereas tremor is not life threatening, it can affect the patient's ability to live comfortable life by compromising various aspects such as dieting, writing, dressing, and self-care. Clinically, tremors are classified as resting tremors, postural tremors, or action tremors [15, 17]. The type of treatment depends on the cause of the tremor; thus, accurate diagnosis of the tremor is paramount. Several parameters such as the amplitude of tremor, subtle changes, efficacy, or fluctuations of tremor cannot be effectively assessed by current clinical evaluation scales [15]. Recent studies show that the misdiagnosis rate of tremor dominant PD or essential tremor is nearly 20–30% [63, 64]. Assessment of tremors is dependent on emotions during the day; thus, short-term regular outpatient follow-ups do not fully reflect the intensity of the tremor [15, 65]. Thus, continuous assessment of the tremors is recommended.

Sensor systems such as accelerometer, gyroscope, goniometer, optical motion capture system, or inertial measurement unit (IMU) have been used to objectively measure PD tremors [15, 19–21, 50, 63, 65–67]. These devices provide long-term monitoring of PD, collection of data related to tremors, as well as identification of the types, and grading the severity of PD [15, 66].

Among all the motion parameters of tremors, wearable devices are used to quantify the frequency of the tremors [15, 17, 65, 66]. Heldman et al. [21] evaluated the performance of a wireless motion sensor unit worn on index finger, based on tasks similar to daily life activities. The data collected showed that spontaneous motion occurred at lower frequency (<3 Hz), compared with tremor which occurred at a frequency of >4 Hz. Similarly, the motion sensor accurately classified the severity of the tremor, thus laying the foundation for accurate categorization of tremors in PD patients. However, the study was performed using a small sample size (10 patients); larger studies were required to obtain more reliable results. In their study, Wile et al. [50] included 41 patients who wore smart watch devices and monitored tremors in 3–6 minutes. The parameters were recorded while the hands were at rest and outstretched. Findings from this study showed that the smart watch was highly specific and sensitive in distinguishing postural reemergent tremors of PD from essential tremor. Thus, smart watch devices are convenient and applicable in the clinical as well as the community settings. However, the monitoring duration (3–6 min) was too short to fully characterize tremors. In a recent study, Braybrook et al. [17] used Parkinson's KinetiGraph System (PKG) to assess the tremors of PD patients and developed a new algorithm by calculating the percentage of tremor time (PTT) presented from 09: 00 to 18: 00 to distinguish resting and postural tremors. This algorithm not only increased the sensitivity and selectivity of evaluating the occurrence of tremors, but also analyzing the relationship between tremors and bradykinesia. In addition, the algorithm identified a threshold at which tremors begin to occur. This approach of quantifying tremors will help researchers to understand the neural mechanisms of PD and develop new therapies.

## 6. Gait

Gait disorder is the main form of postural balance impairment. Often, at the early stages of PD, the swing of upper limbs on the affected side decreases and the lower limbs drags. As PD progresses, freezing of gait (FOG), forward thrust gait, and panic gait develop, all of which make gait disorder the most disabling motor symptom [22, 23]. Impaired dopaminergic signaling increases the risk of gait. Therefore, gait disorder is an emerging surrogate marker of PD progression [26].

Clinicians often use TUG [57–59] to assess gait and observe the balance or flexibility of patients. Whereas postural balance disorder is assessed by the pullback test [68, 69], clinical examination of gait is usually carried out in a broad and well-lit environment, which does not objectively reflect the gait of patients in daily life [70]. Besides, clinical evaluation scales are often limited by the compliance and recall of patients, especially when the patients experience anxiety or depression, making them unable to adequately respond to the questions. It should be noted that movements in patients taking medications vary throughout the day [60]; thus, more effective and objective long-term gait monitoring tools are needed.

The commonly used sensors to detect changes in gait patterns include gyroscope or accelerometer. When placed on different parts of the body (chest, waist, leg, thigh, and foot), these devices provide concise kinematic and dynamic gait parameters to reflect the prognosis of gait [27–29, 37, 47, 59, 71–73]. A number of parameters such as rhythm, symmetry, stride length, amplitude, or periodicity of gait are used to characterize gait and movement of PD patients [38]. This method avoids subjective or brief assessments during clinical visit which improves the accuracy of gait assessment. Wearable sensors not only help clinicians to fully understand the progress of gait disorder, but also provide effective titration of dopaminergic medications. This helps to better evaluate the drug efficacy, the timing of therapy, or adjustment of treatment regiments [24, 30].

A number of studies have conducted objective monitoring of PD gait in experimental conditions or in daily life. For instance, Moore et al. [27] conducted a 24-hour video monitoring of gait using devices worn on the shank. In this way, they continuously monitored and recorded gait while walking and lying down. The results showed the accuracy of gait length monitoring within 24 hours, and the autonomous detection of gait monitor and video observation reached full consistency. Weiss et al. [74] used a triaxial accelerometer on lower back to evaluate gait variability in PD patients before and after taking medication by simulating the activities of daily life. They expected to assess walk quality in real life and discriminate PD patients and healthy subjects. At last, they all verified the feasibility of wearable devices to long-time monitor the gait in laboratory environment and promote the continuous monitoring gait of PD patients in daily life. On the other hand, Del Din et al. [26] explored the impact of the environment and pathology on gait in both laboratory and free-living environment. They used the body-worn monitors (BWMs) to quantitatively assess gait and to compare PD patients and healthy subjects. Results showed that pace, rhythm, asymmetry, and variability of gait in PD patients were increased by daily life activities. They concluded that gait monitoring in living environment is a more sensitive indicator for PD pathology. In addition, if the features of portability, convenience, and accuracy are fully used to provide real-time feedback for gait monitoring, a long-term rehabilitation program for a specific training task could be implemented. Recently, Ferrari et al. [71] created a new zero-velocity-update gait analysis system for real-time gait analysis. This method can be employed to monitor gait and efficacy of interventions anywhere and anytime.

The ability of wearable devices to assess balance and predict risk of falls in PD patients by continuous monitoring of movement in a living environment has been explored in several studies [31, 38, 39, 75–78]. Many of such studies show that sensor devices have higher sensitivity when detecting shorter FOG events, with an accuracy of >90% [32, 33, 79]. Interestingly, such studies found that wearable portable devices can detect gait or balance in real time and provide timely feedback. These devices also provide intelligent reminders through vision, auditory, and touch for PD patients when they experience FOG and falling events. The timely reminders enabled patients to take immediate measures to maintain or improve gait. Taken together, wearable portable devices can prevent the occurrence of relevant events [30, 39, 80], such as FOG and falling events.

## 7. Myotonia

Myotonia is an impairment of muscle relaxation, similar to bending soft lead tube, so called “lead tube” rigidity. Myotonia often presents gear-like rigidity that affects many joints of the body and presents special buckling posture. At the clinical level, the Unified Parkinson's Disease Rating Scale (UPDRS) is often used to evaluate the neck, wrist, elbow, knee, ankle, or other joints of patients [10]. To date, research has focused on quantification of muscle strength through biomechanics. Other methods have also been developed to quantitatively assess myotonia based on the parameters extracted from the passive motion of the joints [81].

Since muscular rigidity is hard to quantify using the current methods, studies have proposed that wearable devices may be effective in quantifying myotonia. For instance, electronic devices, not wearable, have been used to quantify myotonia as a means of revealing neural mechanisms [40]. Another study used a portable transducer to objectively quantify muscle rigidity, and the results were highly consistent with the results of Parkinsonian rigidity clinical score. The specificity and sensitivity of the portable transducer were 89% and 82%, respectively, but only the wrist was quantified [82]. Recently, di Biase and colleagues [83] developed a magnetoinertial motion tracking system to describe the rigidity of joint movement. They placed the sensors on the most affected parts such as the arm, wrist, palm, thumb, or index finger and extracted the total power and smoothness index as parameters that describe the rigidity. They used the tasks of UPDRS III to discriminate off/on motor status as well as PD patients from healthy subjects. Another study quantified axial rigidity through some routine household activities. Phan et al. [68] used BioKin™ consisting of four sensors placed symmetrically on the back of the trunk to record angular velocity signals as well as capture information about instability and stiffness during activities in natural settings. Their study demonstrated that a small number of sensors could monitor variations in rigidity in PD subjects during routine activities. The studies reviewed above indicate that wearable devices are more reliable and feasible in monitoring instability and myotonia in more natural environment.

## 8. Nonmotor Symptoms

With increasing awareness of the presence of nonmotor symptoms in PD, it has been realized that these nonmotor symptoms play an extremely important and sometimes leading role in the treatment and even diagnosis of the disease. Nonmotor symptoms of PD include rapid eye movement (REM) sleep behaviour disorder (RBD), hyposmia, depression, and constipation [84]. Nonmotor symptoms may appear years before those associated with dopamine deficiency or, with other nonmotor symptoms, in the progression of the disease. The neuroanatomical and neuropharmacological basis for nonmotor abnormalities in Parkinson's disease remains largely unknown. More and more attention has been paid to the nonmotor aspects of Parkinson's disease, providing valuable insights into the diversity of the clinical, pathological, and neurochemical characteristics of Parkinson's disease. The nonmotor symptoms and signs of Parkinson's disease are now the subject of effective clinical assessments that can detect their presence and track their progress over time. Wearable devices can monitor nonmotor symptoms of Parkinson's disease, such as RBD, which opens up the prospect of exploring the pathological mechanism of nonmotor symptoms.

Kotschet and colleagues [85] described an algorithmic system of accelerometers placed on the wrist that measures daytime sleepiness. They enrolled 68 PD patients and 30 healthy controls for 10 consecutive days, and somnolence was observed after levodopa administration. The conclusion showed that, compared with the control group, proportion of time immobile (PTI) of PD patients increased 30–60 minutes after taking Levodopa, which confirmed that Levodopa would increase somnolence; PTI can be used as a surrogate measure of daytime sleep in PD patients. McGregor et al. [86] used the Parkinson's KinetiGraph (PKG) as an accelerometry-based system of an objective movement recording system to assess night time sleep, which can provide a static score consistent with sleep. They monitored 155 people older than 60 years with no PD and 72 people with PD for six consecutive days. The results showed that the PKG system had good selectivity (86%) and sensitivity (80%) to distinguish PD patients from healthy controls, and there were significant differences in the scores. This system provides prospects for quantitative sleep scoring in Parkinson's disease patients.

## 9. Prospect

With the development of science and technology, highly sensitive wearable devices have been developed to promote PD research. This has enabled early or differential diagnosis of PD, monitoring of motion state, prevention or reduction of off-stage status, and assessing of movement complications. However, further in-depth research should be carried out on PD because most of the current research has focused on patients with primary PD, typical Parkinson's syndrome, and the main motor symptoms of PD. This will identify more accurate markers of PD progression, nonmotor symptoms of PD, and other types of atypical Parkinson's syndrome [28, 69]. Notably, wearable devices may not be appropriate in cases such as severe motor impairment, off-stage state, cognitive impairment, and for elderly patients [35]. Lastly, it should be noted that wearable devices require unlimited Bluetooth connection for data transmission, and any instability of its connection may lead to data loss [70]. Therefor, there is a need for establishment of a resource sharing platform and enhancement of data protection.

More importantly, in the time of the global COVID-19 outbreak, with the movement of people being limited, patients with Parkinson's disease have not been able to seek medical attention on time, and clinicians have not been able to keep abreast of changes in the patient's condition. At this point, wearables play to their significant advantages, and the telemedicine platform can monitor the motor symptoms and nonmotor symptoms of PD patients. It is helpful for doctors to timely grasp the changes of patients' condition and adjust drug treatment. Wearable devices provide a new approach for the management of Parkinson's disease.
